# Does volunteering decrease burnout? Healthcare professional and student perspectives on burnout and volunteering

**DOI:** 10.3389/fpubh.2024.1387494

**Published:** 2024-05-24

**Authors:** Tai Metzger, Nathan Nguyen, Hillary Le, Daisy Havo, Katherine Ngo, Sebastian Lee, Timmy Nguyen, Quynhanh Nguyen, Leyna Tran, Nathan Tong, Collin Le, Rebecca Dudovitz

**Affiliations:** ^1^Vietnamese Community Health Project, University of California-Los Angeles, Los Angeles, CA, United States; ^2^Oakland University William Beaumont School of Medicine, Rochester, MI, United States; ^3^Division of General Pediatrics, Department of Pediatrics, David Geffen School of Medicine at UCLA, Los Angeles, CA, United States

**Keywords:** COVID-19, volunteering, burnout, community health, medical education, physician shortage, mental health

## Abstract

**Background:**

Burnout among healthcare providers is a significant crisis in our healthcare system, especially in the context of the COVID-19 pandemic. The aim of this study was to understand what motivates healthcare workers and students to volunteer in their community as well as examine how volunteering relates to burnout. These findings can help health organizations better meet the needs of healthcare workers, as well as provide insights for non-profits that rely on volunteer professionals.

**Methods:**

Healthcare providers (*N* = 8), graduate healthcare students (*N* = 10), and undergraduate students (*N* = 14) who volunteered at community health fairs completed the OLBI burnout assessment and an individual semi-structured interview to characterize their attitudes toward volunteering and its relationship with burnout. Interviews were recorded, transcribed, and analyzed using a phenomenological approach, comparing themes across levels of burnout among providers and students.

**Results:**

Participants described that feeling burnt out decreased one’s likelihood to volunteer, but also that volunteering prevented burnout. The OLBI scores showed that 79.2 and 20.8% of students were low and moderately burnt out respectively, and 87.5 and 12.5% of health professionals were low and moderately burnt out, respectively. Students volunteered for professional development while healthcare professionals cited a desire for a change in their day-to-day work as a reason to volunteer. Both students and health professionals often volunteered because they wanted to make a difference, it made them feel good, and/or they felt a responsibility to volunteer. COVID-19 had a wide range of effects on burnout and motivations to volunteer.

**Conclusion:**

Volunteering may be useful for preventing burnout among healthcare workers and students, but may not be helpful for those already experiencing burnout. Interview responses and the fact that none of the volunteers had high burnout levels according to their OLBI scores suggest those who choose to volunteer may be less burnt out. Healthcare organizations and schools can encourage volunteering by emphasizing the difference healthcare students and professionals can make through volunteering in the community. Increasing convenience and emphasizing professional development can help recruit and retain healthcare student volunteers. Highlighting the chance to diversify their scope of practice may help recruit and retain healthcare professional volunteers.

## Introduction

Studies have shown that over 90% of physicians report community participation and promoting health within the community as an important part of their profession (1). However, only 39% of physicians volunteer in this role in a given year (2). Meanwhile, many low-income communities and organizations that serve them depend on volunteer healthcare providers. Understanding what motivates physicians to volunteer is critical to sustaining these efforts.

At the same time, physician burnout is a major problem in our healthcare system, with 62.8% of physicians experiencing burnout (3). It has been shown that burnout often begins during clinical rotations and does not improve throughout or after training (4). Moreover, the COVID-19 pandemic has exacerbated burnout rates and led to staff shortages throughout the United States (5). Despite the extensive research on this topic, solutions to this burnout crisis are not clear (6). While several studies have examined interventions to prevent burnout, volunteering has generally not been considered (7, 8). Moreover, some studies have been done on healthcare students or professionals’ motivations to volunteer, but they do not investigate combating burnout as a potential reason for volunteering. For example, one survey of 286 medical schools in Brazil found that 44% of students volunteered for altruistic reasons, with the others either volunteering as a result of personal duty or academic interests (9). Additionally, in Poland, many students viewed volunteering as part of their civic duty, yet felt unprepared for the staff shortages and heavy workload of the pandemic (10). Thus, altruism may be one lens through which to view the role and effect of volunteering.

A handful of studies document how global health volunteering, including short-term volunteer experiences abroad, might prevent burnout, but we lack an understanding of how local, long-term volunteer experiences relate to burnout (11, 12, 13). In regards to medical students, a 2023 study concluded that global health outreach experiences could potentially reduce burnout (13). One physician volunteer program in Canada reports that younger physicians are easier to recruit as compared to mid-career physicians (14), but few studies have examined how the motivations for volunteering and the role of burnout vary for students who are still in training versus healthcare professionals who have completed training. Given the strain on healthcare workers amid the pandemic, this study delves deeper into their motivations for volunteering and their perception of the role volunteering plays in combating burnout.

To expand on the breadth of the current literature, we included both healthcare students and healthcare professionals, from a variety of fields, such as nursing, medicine, and dentistry. We chose to include healthcare professionals and students from a variety of healthcare fields because past studies on burnout and volunteering have predominantly focused on physicians and to a lesser extent medical students (1, 2, 3, 8, 9, 10, 13, 14). Including more diverse perspectives allowed us to contribute more novel findings to the literature as well as potentially make comparisons between groups. Volunteering was defined as providing free services to a community nonprofit organization offering free healthcare to underserved populations. We sought to characterize student and healthcare professional perspectives on their motivation and attitudes regarding volunteering at local, community-based health fairs, and the relationship between volunteering and burnout.

In summary, the goal of our study was to provide insights about burnout, volunteering, and their relationship from the perspective of students and professionals from various healthcare fields.

## Materials and methods

Vietnamese Community Health (VCH) at the University of California, Los Angeles (UCLA) is a nonprofit that provides free health screenings and services to underserved, predominantly Vietnamese American and Latine, patients through health fairs and other health education and outreach programs. In addition to UCLA undergraduate student volunteers, VCH recruits volunteers from various health professions, including physicians, nurses, pharmacists, chiropractors, and others, as well as students in various health professional schools (e.g., dental). We conducted a concurrent mixed methods study of healthcare professionals and students at various levels of education who chose to volunteer at VCH health fairs. We used both qualitative and quantitative data to describe the perceptions and experiences of the participants in a relatively little-studied area of research (15). Google Sheets and Google Docs were used for qualitative and quantitative analysis. All methods were carried out in accordance with local guidelines and the Declaration of Helsinki. This study was reviewed by the UCLA Human Research Protection Program (OHRPP) and was determined to meet the criteria for exemption from full IRB review on December 15, 2022 (IRB#22–000893).

### Participants

Physicians, medical students, nurses, nursing students, optometrists, optometry students, dentists, dental students, pharmacists, pharmacy students, chiropractic students, acupuncture clinicians, acupuncture students, and pre-health undergraduate students who volunteered at at least one VCH health fair were invited to participate in the study. Participants were contacted via email by a member of the study team or were approached in person at a health fair. Eligibility criteria included having volunteered at one or more health fairs in the past 5 years and being a healthcare professional, healthcare student, or pre-health undergraduate student. All participants were entered into a raffle for four $25 Amazon gift cards. To avoid pressure on volunteers to participate in the study, all potential participants were assured that their participation was completely optional and would not impact their relationship with VCH. We sought to interview participants working in a variety of healthcare fields and in various stages of schooling. Recruitment continued until saturation of major themes and theoretical sufficiency were reached (16, 17).

### Procedure

Data collection occurred from June to December of 2022. Data analysis began in October of 2022 and continued until May of 2023. All potential participants were provided with an information sheet in-person or via email outlining the study methods and goals, as well as potential risks and benefits of participation. The information sheet also included assurances that data would be kept password protected until being permanently deleted after study completion, and that other than the interviews themselves, all data would be de-identified. Only research team members had access to the study documents. After receiving confirmation that participants were interested, participants were scheduled for an interview. Before starting the interview, a member of the research team introduced the study to participants, answered any questions about the study, confirmed eligibility, and obtained oral consent.

After giving oral consent, each participant completed a brief demographic survey and the validated Oldenburg Burnout Inventory (OLBI) assessment to determine their level of burnout (18), which took about 10–15 min to complete in total. The survey and assessment were taken online and were anonymous. This OLBI assessment consists of 16 questions in which respondents select from “Strongly Disagree” (+4), “Disagree” (+3), “Agree” (+2), and “Strongly Agree” (+1). Respondents are classified as “low burnout” if their total is below 44, “moderate burnout” if their total is between 45 and 59, and “high burnout” if their total is 60 or above. “Exhaustion” and “Disengagement” subtotals are calculated as well (19). The OLBI has been accepted to be a reliable and valid measure of burnout in both work and academic settings (20). Participants then completed an individual semi-structured interview via Zoom video conferencing. Interviews lasted 20–60 min and were audio-recorded and transcribed for further analysis.

The interview guide (Table 1) included questions regarding participants’ motivations for volunteering, their experience with burnout, and the impact of burnout and the COVID-19 pandemic on their attitude toward volunteering. Interviews continued until thematic saturation was achieved for both healthcare professionals and students. Themes were continuously identified from the interview transcripts by selected members of the team using a 3-step coding process. Saturation was determined to be complete when subsequent interviews revealed no new codes.

**Table 1:** Interview discussion topics and suggested interview questions.

Discussion topic	Interview questions
Motivations to volunteer	What makes you choose to volunteer at a Health Fair? Probe: What do you get out of volunteering? Probe: What can make your volunteer experience more fulfilling? Probe: How does volunteering at a Health Fair affect your perspective of healthcare?
Effects of the COVID-19 pandemic and burnout on motivation to volunteer	How has the pandemic affected your attitudes toward volunteering? What role does burnout or emotional exhaustion caused by your career play in your decision to volunteer? How do you combat burnout? How has the pandemic affected your attitudes toward burnout?
Effect of role in healthcare on motivation to volunteer	*If a student:* How does your role as a student affect your attitude toward volunteering? Probe: How do you expect your attitude toward volunteering to change after you graduate and work as a healthcare professional? Probe: What do you think could be done to encourage more students to volunteer? *If a healthcare professional:* How does your role as a healthcare professional affect your attitude toward volunteering? Probe: How is your attitude toward volunteering different now compared to when you were a student? Probe: What do you think could be done to encourage more healthcare professionals to volunteer?
Open-ended discussion	Is there anything else you would like to share regarding the personal impact volunteering has had on you, in general or during the pandemic?

### Data analysis

Throughout the interview process, interviewers met to discuss emerging themes and develop and refine the codebook.

Qualitative analysis was conducted using a 3-step coding process based on grounded theory and thematic analysis (21). Three coders reviewed transcripts and coded them using the codebook. They discussed and refined the codes until a Kappa greater than 0.8 was achieved for all major codes. Personal opinions, beliefs, and judgments of the coders were omitted as much as possible when coding the interview transcripts in order to ensure reliable results. Interviews were then divided among the coders to complete the coding process. The analytic team then discussed and identified connections between codes and themes that emerged. Perspectives on volunteering were compared across levels of training and burnout levels.

The quantitative data from the OLBI assessment and demographic survey were also analyzed using unpaired t-tests to describe our sample and compare differences between groups. Burnout levels were compared across levels of training, sex, age, and country of birth.

## Results

Overall, 32 interviews were conducted with 14 undergraduate students, 10 graduate healthcare students, and 8 healthcare professionals. Summaries of participant demographics and main themes with representative quotations can be found in Tables 2 and 3 respectively. Our interviewees’ ages ranged from young adults (18–24) mainly for undergraduate and graduate students to older adults (45-older) for healthcare professionals. The proportion of males to females was nearly equal, with 46.9% being males and 53.1% being females. The majority of our participants identified as Asian/Pacific Islanders and were born in the United States.

**Table 2:** Participant demographics.

	Undergraduate volunteer (*N* = 14)		Graduate healthcare student (*N* = 10)		Healthcare professionals (*N* = 8)	
	Number	Percent	Number	Percent	Number	Percent
*Age*						
18–24	14	100%	5	50%	0	0%
25–34	0	0%	4	40%	1	12.50%
35–44	0	0%	0	0%	0	0
45–54	0	0%	1	10%	3	37.50%
55 or older	0	0%	0	0%	4	50%
Sex						
Male	7	50%	4	40%	4	50%
Female	7	50%	6	60%	4	50%
*Race/Ethnicity*						
Asian/Pacific Islander	14	100%	5	50%	7	87.50%
Non-Hispanic White	0	0%	3	30%	1	12.50%
Asian/Pacific Islander and Non-Hispanic White	0	0%	3	10%	1	0%
Hispanic	0	0%	1	10%	0	0%
*Born in the US*						
Yes	13	92.90%	9	90%	3	78.10%
No	1	7.10%	1	10%	5	21.90%
*Average OLBI Scores*						
Disengagement Subtotal	18.57		16.1		15.38	
Exhaustion Subtotal	21.07		22		15.25	
OLBI Total Score	39.64		38.1		30.63	

**Table 3:** Themes and representative quotations from healthcare professionals and students.

Themes	Subthemes	Example quotes
Motivations to Volunteer	(1) Intrinsic motivations (Students and Professionals)	Pre-health student: “it’s very satisfying to know … the work you put in has directly helped the world and you did it for free.” Physician: “If I can encourage someone to take better care of their health, I feel that with my role as a healthcare professional with years of experience, hopefully, makes some sort of impact in the person’s life.” Optometry student: “I feel that…it’s my responsibility” Student: “So It just makes me feel good. I mean like, it’s exhausting but it makes you feel good.” Nurse Practitioner: “It makes me feel good to be able to give back and to be part of educating people about their health”
	(2) Professional development (Students)	Pre-health student: “I volunteer at places because I believe in the mission…But at the same time…I know it’s going to be on my resume or on an application.” Pre-health student: “Volunteering can give a lot of experience clinically, so I think that’s the main reason I volunteer at the Health Fairs because they are a good source of experience for me” Medical student: “I would not say that community health is one of my interests at the moment, but I am interested in matching to a good residency in the future. So, unfortunately, there are boxes to check, so to speak, and so getting involved in the community and showing involvement with people ranks very highly with programs.” Pre-health student: “[I] want to volunteer more to put my knowledge and the thing I learn in class into practice.” Pre-health student: “So I guess volunteering is a really good way to apply the things you learned.” Pre-health student: “I enjoy…working with healthcare professionals, I feel like a lot of good insight they can give me, especially when it pertains to applying to med school and working as a physician. I feel like I have a lot to learn from them, so it’s great that I can work so closely and get good advice from them”
	(3) Diversifying and expanding on their day-to-day work (Professionals)	Physician: “for the past you know 10 years my work has taken me away from direct contact with the community so it was a nice way to re-engage with the patient’s um Vietnamese patients and also the students.” Physician: “I enjoyed volunteering because it provides me with a change in daily activity.”
	(4) Mentoring the next generation (Professionals)	Nurse Practitioner: “For me, it’s to give my students the opportunity to volunteer. I think it’s a good experience for them.” Physician: “I participate in these kinds of activities because…it is great to see young people participating and being engaging…” Physician: “The desire to teach the next generation…”
Students shared similar perspectives on volunteering in the future	(1) Volunteer altruistically instead of for personal gain	Pre-health student: “Once I’m established as a professional, I think for me volunteering would become less of oh I want to do it for my resume and would become more like I want to do it just because I really care about this organization…And once we have gotten past all that and have become established as individuals within our career, we can really separate ourselves from the whole I need to do things pertaining to my field. Because at least for me personally, I would love to volunteer at more things unrelated to my field just because I’m interested in it.”
	(2) Looking forward to being able to make more of a difference	Pre-health student: “As a healthcare professional, just being able to like do more for the patient…I feel like it could be more rewarding to like volunteer like that.”
	(3) Try to volunteer in different settings outside of healthcare	Pre-health student: “I would say that like if I were to become a healthcare professional or like after graduation, I would say my attitude toward volunteering would still be largely the same, just like trying to help others. But maybe just find different avenues to help people like since I would already be a “healthcare professional,” I could maybe volunteer at a soup kitchen or something like that.”
The bidirectional relationship between volunteering and burnout	(1) Burnt out professionals and students are less motivated to volunteer	Pre-health student: “burnout is definitely real and it affects my decision to volunteer by making me less likely to want to volunteer.” Pre-health student: “[burnout] acts as like a limiting factor so especially this quarter I really did not do as much hospital volunteering.” Physician: “if people feel burnout they may not want to volunteer.”
	(2) Volunteering combats burnout for both students and professionals	Pre-health student: “I think [volunteering] kind of helps the burnout because…it kind of reminds me of what I want to do in the future and like eases the burnout a little because when I’m doing school I’m not like you know seeing a lot of impact in the community.” Nurse Practitioner: “…I think for me it actually combats burnout or emotional exhaustion …” Physician: “I think if you can help people and then they tell you that you are helping them tremendously, then you will not get burnout.”
Barriers and facilitators to volunteering	(1) Increasing convenience and accessibility	Pre-health student: “One of the things is how easy you guys make it. Because like if you do not have the transportation you will provide it. I think that is huge because otherwise a lot of people do not have that opportunity.” Chiropractic student: “I think largely marketing. If you have posters or flyers that we could share with people, that would be pretty cool.” Physician: “I think just make it more convenient for them”
	(2) Consideration of Individual Preferences	Pre-health student: “I think just being mindful of preferences I think is the biggest thing” Physician: “I think you do give people food…I thought it was good that you send out certificates and thank you notes”
	(3) Showing impact to foster intrinsic motivation	Optometry student: “Having patient testimonials, the reasons why they enjoyed it or things like that to see the benefit is appreciable and they can actually make an impact on these communities” Physician: “I think all of us have to work on telling [potential volunteers], “Hey even if you can even help one person, or teach one kid, or show that one kid direction, or show that one patient that hey, there’s someone here who can at least listen to you for 5 min, I think you have done a lot.”
	(4) Making Friends/Socialization	Pre-health student: “For me, the most important thing is probably just like making friends or making connections that I feel are really impactful for my life.”
Effects of COVID-19 on volunteering and burnout	(1) The pandemic increased burnout for some individuals	Pre-health student: “I feel like the pandemic kind of magnified the effects of burnout.” Nurse Practitioner: “In terms of burnout in general, the pandemic was a really difficult time.”
	(2) The pandemic decreased burnout for some individuals	Pre-health student: “During the pandemic, I do not think I experienced as much burnout” Physician: “The pandemic rejuvenated or revived our attitudes and broke the monotony of our practice that may contribute to burnout for some people.”
	(3) The pandemic decreased opportunities for volunteering, but increased motivation to volunteer	Pre-health student: “The pandemic definitely limited my opportunity and my time and access to more volunteer, but increased my need and want to volunteer more to make up for the lost time.” Physician: “I had always had a strong sense of wanting to participate in activities…during the pandemic, when we were unable to do that, I felt that there were many people who needed us, but were not able to reach out.”
	(4) Fear of spreading COVID-19 while volunteering	Chiropractic student: “You’re going to be in a public space…there’s a possibility that you get it from patient B if you are right next to them…The question is how do we do it [volunteer] as safely as possible?” Nurse Practitioner: “When the numbers were higher, I definitely did not want to volunteer.”

According to the OLBI Assessments that our participants completed, 79.2 and 20.8% of students were low and moderately burnt out respectively, and 87.5 and 12.5% of health professionals were low and moderately burnt out respectively. The standard deviation of student OLBI scores was 5.7 and the standard deviation of professional OLBI scores was 10.9, suggesting that there may be wider variance in levels of burnout among health professionals than health students. Women had a higher average total OLBI score than men (*p* = 0.15), students had a higher average total OLBI score than health professionals (*p* = 0.07), and those born in the United States (U.S.) had a higher average total OLBI score than those born outside the U.S. (*p* = 0.27), though no differences reached statistical significance. None of the participants had high levels of burnout. Due to the small sample size, these quantitative results are primarily presented to provide descriptive information about our sample (see Tables 4, 5).

**Table 4:** OLBI scores by sex.

	Men	Women
Disengagement Subtotal (*p* = 0.50)	16.5	17.4
Exhaustion Subtotal (*p* = 0.09)	18.2	21.4
OLBI Total Score (p = 0.15)	34.7	38.8

**Table 5:** OLBI score comparisons by demographic.

	Average OLBI total scores ± Standard deviation
Student Professional (*p* = 0.07)	39.0 ± 5.7 30.6 ± 10.9
Male Female (*p* = 0.15)	34.7 ± 7.1 38.8 ± 8.5
Born in US Born Outside (*p* = 0.27)	38.0 ± 6.0 32.9 ± 12.8

**Figure 1:**
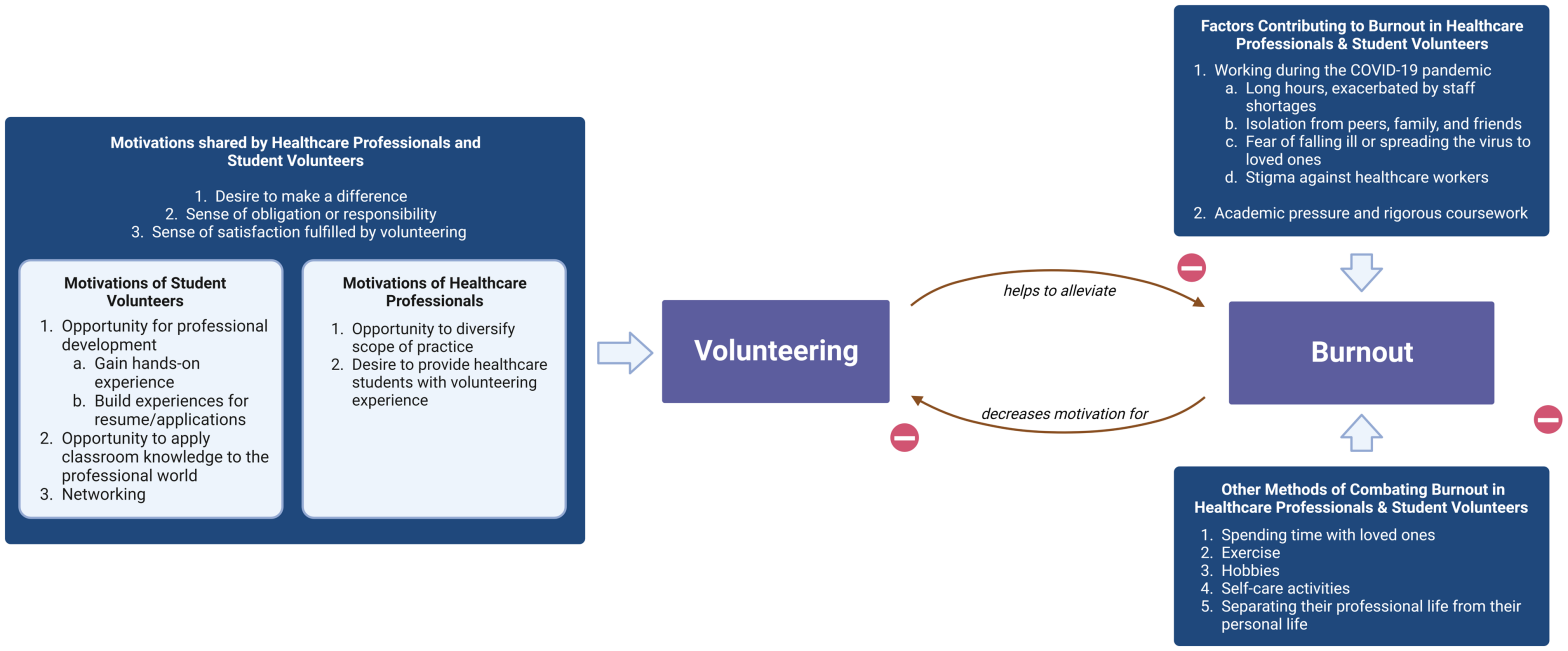
Healthcare professionals and student volunteers demonstrated a wide range of motivations to volunteer, commonly citing the desire to make a difference and their sense of obligation and satisfaction fulfilled through volunteering. Participants expressed that volunteering, amongst other factors, helped to alleviate burnout caused by the COVID-19 pandemic and by academic pressure. However, participants also indicated that burnout decreased their motivation to volunteer.

Qualitative analysis revealed five main themes: motivations to volunteer, shared student perspectives on volunteering in the future, the bidirectional relationship between volunteering and burnout, facilitators and barriers to volunteering, and the effects of COVID-19 on volunteering and burnout. “Motivations to volunteer” refers to the reasons participants cited as making them desire to volunteer, such as professional development for students, diversifying their daily activities for professionals, and intrinsic feelings of responsibility felt by both students and professionals. “Shared student perspectives on volunteering in the future” reflected how students predicted what their volunteering would look like after completing their training. For instance, many students stated that they expected to continue volunteering in the future because they would be able to make more of a difference after completing more training. “The bidirectional relationship between volunteering and burnout” refers to participant descriptions of how burnout decreased motivation to volunteer while volunteering protected against developing burnout. “Facilitators and barriers to volunteering” describes the factors that would make participants more or less likely to engage in volunteering, such as flexibility being a facilitator and inconvenient locations being a barrier. “The effects of COVID-19 on volunteering and burnout” refers to the role of the COVID pandemic as either increasing or decreasing feelings of burnout as well as enhancing or reducing the likelihood of volunteering, depending on the individual. Within each of these overarching themes, sub-themes were established. The themes and sub-themes are presented in Table 3. Each sub-theme represented a key finding related to its associated theme. Example quotes displaying each sub-theme have been provided in Table 3. While the main themes were present in all interviews, several sub-themes differed between students and professionals. Figure 1 encompasses the broad findings from these interviews. Representative quotations found in Table 3 and the following paragraphs provided insight into the qualitative data highlighting the perspectives of the participants in regard to burnout and volunteering.

Demographic characteristics did not appear to relate to noticeable differences in perspectives on volunteering and burnout.

### Motivations to volunteer

Both students and health professional volunteers discussed their desire to make a difference for those in need as an important motivation for volunteering. As one student stated, “I also want to contribute some effort and time to help the community that is in need of help.” This desire to make a difference was sometimes expressed as an obligation or responsibility to volunteer. For example, when asked why they volunteer, one nurse answered, “As a healthcare professional and more specifically as a nurse, I think it’s part of my responsibility to the community to be able to volunteer.” In addition, participants described how volunteering and positively impacting others made them feel good or fulfilled. For example, an optometry student said they choose to volunteer because they “get a great feeling to help people.”

There were some motivations specific to student volunteers. Students described volunteering for professional development. For instance, some interviewees wanted to gain hands-on experience in the healthcare field, while others explicitly stated that it would be useful for their resumes or applications. Similarly, some students mentioned that volunteering in a healthcare setting allowed them to apply what they learned in class to real-life situations, such as one student who said, “I chose to volunteer because I thought it was a good opportunity to practice my skills outside of school.” Finally, a few students discussed volunteering in order to network with health professionals or older students.

In contrast, health professionals described volunteering because the experience provided a change in their normal day-to-day work, allowing them to diversify and expand on their usual scope of practice and career. One participant said, “I think that, out of the 9–5, the very routine kind of nature of optometry, for example, it’s kinda nice to really reach out and you feel very good that you are helping the community, and you are normally, but it’s outside your normal, everyday day-to-day same office, same place, same people.” Additionally, several health professionals, particularly the preceptors overseeing their student volunteers, cited wanting to give their students more opportunities to gain experience through volunteering. They also found it inspiring to interact with future health professionals. One physician stated, “Seeing and watching how engaged the young people are, it gives me a good feeling and a lot of hope…I find that it is an extremely meaningful use of my time.”

Lastly, while students tended to think that their attitudes and reasons for volunteering might change after becoming health professionals, most health professionals reported their attitude toward volunteering was similar to when they were students. Specifically, students expressed that they expected to be able to volunteer for more fully altruistic reasons rather than for gaining experience and that they looked forward to being able to make more of an impact through their volunteering when they completed their training. Some students also expressed interest in volunteering outside of healthcare in order to explore fields unrelated to their career goals.

### Bidirectional relationship between volunteering and burnout

Participants described a complex and bidirectional relationship between burnout and volunteering, whereby being burnt out would make one less likely to volunteer, and volunteering reduced burnout when the volunteer did not already feel burnt out. For example, one volunteer said, “[Burnout] definitely makes me not want to volunteer because it takes up time of your personal life and when you already have a lot of work to do.” Meanwhile, participants also described how volunteering helped both students and health professionals reinvigorate their motivation for pursuing a career in healthcare. One student said: “It makes me more connected to my goals of being a doctor because you are actually seeing healthcare in action and not just studying all day.” Similarly, one physician volunteer said that volunteering helped “renew my interest in and remembering why I had gone into medicine in the first place.” These themes were consistent across students and health professionals and across those with low versus moderate burnout scores.

### Facilitators and barriers to volunteering

One method of incentivizing health professional and student volunteers is the convenience of and accessibility to volunteering opportunities. Transportation in particular was frequently noted as a barrier by students. Additionally, the consideration of individual preferences was seen as a positive by student volunteers. Participants suggested showcasing the difference made by volunteering in the community to foster intrinsic motivation and encourage greater student and health professional participation in events. For example, one member suggested that previous volunteers “tell their story, and maybe show videos or show different forms of media of how this opportunity has impacted a patient’s life because I feel you can only say so much, and you just have to hear it and, like, see it from someone.” to help with recruitment. A healthcare professional explained, “hearing positive stories or success stories from the patients as a feedback to the caregiver can be very motivating for them to continue to show up.”

Additionally, participants suggested socialization or making friends facilitated volunteer recruitment and retention. As one student volunteer stated, “when you volunteer you are in an environment working together either over multiple sessions or just for a longer amount of time, so that gives you more opportunity to get to know people.” A significant barrier to volunteering for both students and health professionals was a lack of time or energy caused by work, studies, and/or other demands.

### Effect of COVID-19 on burnout and volunteering

Participants reported a wide range of effects of the COVID-19 pandemic on burnout. For some individuals, the pandemic increased the amount of burnout, whereas for others, it had no effect or even a positive effect. One participant stated that “the pandemic definitely puts more strain on physicians and healthcare providers so it certainly can make burnout more challenging, more prevalent, [and] more prominent.” Similarly in education settings “it was harder to connect with the students… [and] it was harder for them because they did not have each other to lean on” which made “burnout… a really big deal… during the pandemic.” Across all settings, volunteers discussed how social isolation and the lack of in-person activities during the pandemic was a challenge that contributed to burnout.

In addition, many individuals described losing a sense of freedom and routine they were accustomed to. COVID-19 also introduced new sources of stress as individuals felt that “it’s become a lot [easier] to get burned out” because [of the tension] around healthcare… [as] they are wondering if [they] have COVID.”

In contrast, COVID-19 sometimes had opposite effects on individuals’ feelings of burnout. For example, one provider described feeling a “sense of urgency that people’s health were at risk and that [they] had to do something new.” Additionally, another individual mentioned how they did not “feel burned out because…it was a lot slower during the pandemic.”

The COVID-19 pandemic also had varying effects on the participants’ decision to volunteer. For some, the heightened healthcare needs in the community prompted them to want to volunteer more once they were able to, such as one participant who stated, “The pandemic made me feel that once things were opening again, that there was really a strong need for being able to provide these services.” Alternatively, concern about contracting COVID-19 deterred some participants from volunteering during the pandemic. Participants also generally wanted volunteering opportunities to return to pre-pandemic levels and hoped for a return to normalcy.

## Discussion

### Key findings

Our study had several key findings. First, the results suggested that while burnout is a barrier to volunteering, participants thought volunteering helped them avoid burnout. Rather than reducing burnout in individuals who feel emotionally exhausted or disengaged from their work, volunteering seems to be more effective at preventing these feelings from occurring in individuals who are at risk for–but not yet experiencing–burnout. In addition, healthcare professionals and students may have some different motivations for volunteering, but feelings of gratification and a sense of responsibility to the community remained consistent for both groups, as well as the desire to make a difference. Students specifically volunteered to gain experience and advance their professional goals, while health professionals cited a desire for experiences different from their daily work activities. Furthermore, burnout amidst the COVID-19 pandemic and transitioning out of the pandemic affected individuals differently with some choosing to volunteer to combat burnout while others became more reluctant to volunteer. This is the first study analyzing motivations for volunteering along with the role of burnout across training levels of healthcare professionals and students. Ultimately, these findings can guide healthcare organizations in combating burnout as well as recruiting and retaining volunteers.

### Burnout and volunteering

The fact that none of the volunteers interviewed in this study had high levels of burnout is consistent with this reciprocal relationship between burnout and volunteering. Our findings are also consistent with studies regarding physician volunteering in global health settings suggesting physicians experienced gratification from being able to help patients with difficult circumstances and insufficient resources as well as from being able to contribute to teaching and learning opportunities during medical missions (22). In addition, volunteering on mission trips improved mental health and decreased burnout scores (11–13). Thus, our study suggests some of the benefits of international service trips are also present in local volunteering opportunities, which may be both more accessible and sustainable than global health programs.

Previous studies that examined motivations for volunteering within the healthcare community primarily focused on physicians, particularly in foreign countries. A study from 2017 found that many physicians volunteered due to “humanitarian and prosocial desires,” and wanted to practice medicine in a setting without the stress and demands of their daily routines (23). Another study investigated the motivations of those who participated in short-term medical missions, sometimes referred to as “voluntourism,” and concluded that personality is a major factor in one’s decision to volunteer abroad (22). Our findings build on this work. By examining the relationship between volunteering and burnout more broadly and including a variety of health professionals and students, we provide a more holistic view of volunteering perspectives within the healthcare community. Our present study also allows a comparison between healthcare volunteering internationally versus locally, showing that similar benefits to the volunteers are present in both.

Based on the interview responses, we hypothesize several aspects of volunteering that may contribute to reduced burnout. First, it was clear that the participants were motivated by seeing the positive impact of volunteering on those in need. Presumably, many of the participants entered or are planning to enter the healthcare field due at least in part to a desire to improve the health of others. Thus, volunteering may reduce burnout by providing a clear reminder of their purpose and original desire to work in healthcare. This hypothesis is consistent with the Social Cognitive Theory of causes of burnout that posits that burnout could be due to doubts about one’s effectiveness, ability to achieve goals, and lack of reinforcement for their work (24). Moreover, our findings support the Social Exchange Theory of burnout which states that the disconnect between the results from one’s work and the efforts put into the work, as well as a lack of reciprocity, could lead to emotional exhaustion and burnout (24). Volunteering could alleviate the perceived lack of reciprocity and sense of impact when the recipients and hosting organization show their strong appreciation. This sentiment of desiring to know their service was valued was evident in the quotes highlighted previously.

### The role of COVID-19

In addition, we build on previous findings by describing a wide variety of perceived effects of COVID-19 on volunteering and burnout. COVID-19 seemed to have increased burnout for some and decreased burnout for others, supporting the idea that the pandemic’s influence on individuals’ perceptions of burnout was diverse. The pandemic served as a facilitator for volunteering by reinforcing the acute need for healthcare professionals in some cases but acted as a barrier to volunteering in other cases by creating the fear of contracting and spreading the virus.

### Limitations and future research directions

This study had some limitations. To begin, all of the undergraduate students we interviewed were from the University of California, Los Angeles (UCLA), and are from the same organization, Vietnamese Community Health (VCH), which limits the generalizability of our findings. The fact that none of the participants were found to have high levels of burnout also limits the study by preventing us from learning about the perspective of individuals with high burnout levels (although this may provide evidence that individuals with high levels of burnout are less inclined to volunteer). In addition, although participants were assured of confidentiality and de-identification of their interviews and OLBI results, responses may be limited by social desirability response bias, particularly with regards to feelings of burnout, which can be a personal subject, and motivations to volunteering (25). Lastly, the participants were disproportionately Asian/Pacific Islander, possibly reducing how representative these volunteers may be of the general student and healthcare worker populations.

Future research studies can address these limitations by exploring these findings in other geographic areas and demographic groups to expand generalizability. For more specific quantitative results, a larger sample size can be studied, allowing for better comparisons about burnout and volunteering between different demographic groups, levels of training, and healthcare fields. This would allow for a better understanding of the various motivating factors specific to different subgroups. Future studies can also study the effects of implementing the interventions recommended in Table 6.

**Table 6:** Recommendations.

	For schools and healthcare organizations to decrease burnout:	For non-profit organizations and volunteer programs to retain and recruit volunteers
Recommendations:	Provide volunteer opportunities for students and healthcare professionals to gain skills (students) and prevent burnout (students and professionals) Do not pressure individuals suffering from burnout to engage in volunteering For healthcare organizations, highlight the impact of the efforts made by staff by providing patient testimonials expressing appreciation Increase opportunities for having personal connections with others	Emphasize the concrete difference made by the organization’s work Advertise specific stories of how service recipients have been impacted positively by volunteering For students, highlight professional and career skills development For healthcare professionals, highlight the unique experiences available through volunteering Include opportunities for creating friendships and connecting with others Make volunteer opportunities flexible and convenient Appeal to feelings of responsibility to specific communities in need

### Implications and recommendations

Despite these limitations, these findings have important implications that may be useful in combating burnout as well as recruiting and retaining volunteers. Specifically, healthcare organizations might consider promoting volunteering as a possible method of preventing burnout and exhaustion among their employees so that they can gain experiences outside of their usual work and rekindle their original motivations for entering the healthcare field by helping those in need. Universities and health professional schools might consider formally integrating volunteer experiences into curricula to both prevent burnout as well as to provide meaningful, educational, and professionally enriching experiences outside of the classroom. Since burnt-out individuals are less likely to volunteer, these volunteer programs should be implemented while the students or professionals are not feeling burnt out. In other words, volunteering may be an effective preventive method against burnout but a poor, or even counterproductive, intervention for those who are already experiencing burnout. As the first study of this kind exploring relatively novel concepts, this paper can provide a starting point for generating research questions for additional studies. Table 6 provides a summary of proposed recommendations for healthcare organizations, schools, and volunteer programs.

Lastly, non-profit healthcare organizations that rely on volunteers might use these findings to recruit and retain health professionals and student volunteers. For health professionals, for example, it may be beneficial to emphasize the opportunity to gain experiences that professionals normally do not get in their typical practice, while for students, opportunities for professional development could be emphasized. Additionally, organizations should consider showcasing their impact through patient testimonials, make efforts to increase the convenience and flexibility of volunteering opportunities, and appeal to individuals’ intrinsic motivation to volunteer by highlighting their responsibility to the community. Indeed, seeing that their volunteering directly made a difference in the lives of those in need was overall viewed as the most important factor in deciding to volunteer.

## Data availability statement

The datasets presented in this article are not readily available because the main sources of data for this study were video interviews so we are unable to make this data publicly available. Some material may be made available by the authors upon request from interested researchers. Requests to access the datasets should be directed to taimetzger@g.ucla.edu.

## Ethics statement

This study was reviewed by the UCLA Human Research Protection Program (OHRPP) and was determined to meet the criteria for exemption from full IRB review on December 15, 2022 (IRB#22–000893). The studies were conducted in accordance with the local legislation and institutional requirements. The Ethics Committee/Institutional Review Board waived the requirement of written informed consent for participation from the participants or the participants’ legal guardians/next of kin because there was no risk of harm to participants (just a survey and interview were utilized using the internet) so oral consent could be obtained instead of written informed consent.

## Author contributions

TM: Conceptualization, Data curation, Formal analysis, Funding acquisition, Investigation, Methodology, Project administration, Resources, Software, Supervision, Validation, Visualization, Writing – original draft, Writing – review & editing. NN: Conceptualization, Data curation, Formal analysis, Funding acquisition, Investigation, Methodology, Project administration, Resources, Software, Supervision, Validation, Visualization, Writing – original draft, Writing – review & editing. HL: Conceptualization, Data curation, Formal analysis, Investigation, Methodology, Writing – original draft, Writing – review & editing. DH: Conceptualization, Data curation, Formal analysis, Investigation, Methodology, Writing – original draft, Writing – review & editing. KN: Conceptualization, Investigation, Methodology, Visualization, Writing – original draft, Writing – review & editing. SL: Conceptualization, Investigation, Methodology, Writing – original draft, Writing – review & editing. TN: Conceptualization, Data curation, Formal analysis, Investigation, Methodology, Writing – original draft, Writing – review & editing. QN: Investigation, Methodology, Writing – original draft, Writing – review & editing, Conceptualization. LT: Conceptualization, Investigation, Methodology, Writing – original draft, Writing – review & editing. NT: Conceptualization, Investigation, Methodology, Writing – original draft, Writing – review & editing. CL: Conceptualization, Investigation, Methodology, Writing – original draft, Writing – review & editing. RD: Conceptualization, Data curation, Formal analysis, Funding acquisition, Methodology, Project administration, Resources, Supervision, Validation, Writing – original draft, Writing – review & editing.
